# Accumulating Sedentary Time and Physical Activity From Childhood to Adolescence and Cardiac Function in Adolescence

**DOI:** 10.1161/JAHA.123.031837

**Published:** 2024-03-16

**Authors:** Eero A. Haapala, Marja H. Leppänen, Earric Lee, Kai Savonen, Jari A. Laukkanen, Mika Kähönen, Soren Brage, Timo A. Lakka

**Affiliations:** ^1^ Faculty of Sport and Health Sciences University of Jyväskylä Jyväskylä Finland; ^2^ Institute of Biomedicine, School of Medicine University of Eastern Finland Kuopio Finland; ^3^ Faculty of Medicine University of Helsinki Finland; ^4^ Foundation for Research in Health Exercise and Nutrition Kuopio Research Institute of Exercise Medicine Kuopio Finland; ^5^ Institute of Public Health and Clinical Nutrition University of Eastern Finland Kuopio Finland; ^6^ Department of Medicine Wellbeing Services County of Central Finland Jyväskylä Finland; ^7^ Department of Clinical Physiology, Tampere University Hospital and Faculty of Medicine and Health Technology Tampere University Tampere Finland; ^8^ MRC Epidemiology Unit University of Cambridge School of Clinical Medicine Cambridge United Kingdom; ^9^ Department of Clinical Physiology and Nuclear Imaging University of Eastern Finland and Kuopio University Hospital Kuopio Finland

**Keywords:** exercise, heart function, pediatrics, sedentary behavior, Exercise, Pediatrics, Epidemiology, Lifestyle

## Abstract

**Background:**

Increased physical activity (PA) may mitigate the negative cardiovascular health effects of sedentary behavior in adolescents. However, the relationship of PA and sedentary time from childhood with cardiac function in adolescence remains underexplored. Therefore, we investigated the associations of cumulative sedentary time and PA from childhood to adolescence with cardiac function in adolescence.

**Methods and Results:**

Participants were 153 adolescents (69 girls) who were aged 6 to 8 years at baseline, 8 to 10 years at 2‐year follow‐up, and 15 to 17 years at 8‐year follow‐up. Cumulative sedentary time and PA exposure between baseline and 2‐year follow‐up and between baseline and 8‐year follow‐up were measured using a combined accelerometer and heart rate monitor. Cardiac function was assessed using impedance cardiography at 8‐year follow‐up. The data were analyzed using linear regression analyses adjusted for age and sex. Cumulative moderate to vigorous PA (standardized regression coefficient [β]=−0.323 [95% CI, −0.527 to −0.119]) and vigorous PA (β=−0.295 [95% CI, −0.508 to −0.083]) from baseline to 8‐year follow‐up were inversely associated with cardiac work at 8‐year follow‐up. Conversely, cumulative sedentary time had a positive association (β=0.245 [95% CI, 0.092−0.398]). Cumulative vigorous PA from baseline to 8‐year follow‐up was inversely associated with cardiac work index at 8‐year follow‐up (β=−0.218 [95% CI, −0.436 to 0.000]).

**Conclusions:**

Higher levels of sedentary time and lower levels of PA during childhood were associated with higher cardiac work in adolescence, highlighting the importance of increasing PA and reducing sedentary time from childhood.

**Nonstandard Abbreviations and Acronyms**
MVPA: moderate to vigorous physical activity
PAEE: physical activity energy expenditure
PANIC: Physical Activity and Nutrition in Children
PWV: pulse wave velocity

**Clinical Perspective**

**What Is New?**

- Increased levels of sedentary behavior raise the risk of cardiovascular diseases, but little is known about the role of sedentary time and physical activity in cardiac work and function in youth.
- We found that adolescents accumulating higher levels of sedentary time and lower levels of physical activity from childhood had higher cardiac workload compared with their more physically active peers. However, these associations were partly explained by adiposity and other cardiometabolic risk factors.

**What Are the Clinical Implications?**

- These findings highlight the importance of promotion of a physically active lifestyle and obesity prevention and weight management from childhood to prevent abnormalities in cardiac function later in life.

Adolescents are adopting increasing amounts of sedentary behaviors,^1^ with <20% of them achieving the recommended levels of moderate to vigorous physical activity (MVPA).^2^ Increased time spent in sedentary behaviors, defined as “any waking behaviors spent sitting, reclining, or lying postures with low energy expenditure,”^3^ may increase the risk of atherosclerotic cardiovascular diseases.^4^ Conversely, increased physical activity (PA) has been suggested to counteract the undesirable effects of sedentary behavior on cardiovascular health.^5^ However, the association of sedentary behavior from childhood with cardiac work and cardiac function in adolescence remains poorly understood.

Although studies among adults suggest that prolonged bed rest negatively alters cardiac structure and function,^6^, ^7^ observational studies have reported weak associations between sedentary time and cardiac structure or function.^8^, ^9^, ^10^ In contrast, higher levels of MVPA and vigorous PA have been associated with better cardiac functions in adults.^10^, ^11^ Additionally, high‐ and moderate‐intensity interval training have been associated with improved resting cardiac function.^12^, ^13^ Regular PA has also been associated with reduced myocardial workload in adults.^13^ In children and adolescents, however, the role of sedentary time on cardiac work and function has received far less attention.^14^, ^15^ Furthermore, the associations of PA from childhood to adolescence with cardiac work and function in adolescence has yet to be explored. Cardiac work is closely correlated with cardiac oxygen consumption and could be an important preclinical marker of cardiac overloading in youth. As such, it could provide early‐stage information on left ventricular wall stress and risk of left ventricular hypertrophy and heart failure.^16^

Nevertheless, the mechanisms explaining the associations of sedentary time and PA with cardiac work and function in youth remain unexplained.^13^, ^17^, ^18^ However, lower cardiac function has been associated with reduced levels of circulating high‐density lipoprotein (HDL) cholesterol.^19^ Higher serum HDL particle levels may contribute to cardiac functions by reducing myocardial hypertrophy induced by increased left ventricular wall stress, decreasing cell injury and regulating intracellular signaling pathways.^20^ Moreover, a sedentary lifestyle and physical inactivity have been found to induce insulin resistance, increase blood pressure, and arterial stiffness, potentially increasing cardiac work and impairing cardiac function.^13^, ^17^, ^18^

Most pediatric studies on the associations of sedentary time and PA with cardiovascular health have been cross‐sectional or have included only a short follow‐up period.^15^, ^21^ In addition, a majority of these studies have focused on arterial structure and function and have not explored potential mechanisms or modifying factors for these associations. Time spent in sedentary behaviors and PA during childhood could carry over into adolescence.^22^, ^23^ The change in cumulative sedentary time and PA over this crucial developmental period may also affect cardiac work and function. Given that this is an area that has yet to be fully elucidated, we examined the relationship of cumulative sedentary time and PA from childhood to adolescence with cardiac work and function in adolescence over an 8‐year follow‐up period. Moreover, because sedentary time and PA may have differing effects on cardiac functions during different development periods,^24^ we also investigated whether cumulative sedentary time and PA in childhood between baseline and 2‐year follow‐up were associated with cardiac work and function at 8‐year follow‐up.

## METHODS

The data that support the findings of this study are available from the corresponding author upon reasonable request.

### Study Design

The present longitudinal cohort data are from the PANIC (Physical Activity and Nutrition in Children) study, which is an 8‐year physical activity and dietary intervention study and a long‐term follow‐up study in a population sample of children from the city of Kuopio, Finland.^25^ The main analyses included participants who had valid data on cardiac work and function at 8‐year follow‐up and valid sedentary time and PA data at least at 1 time point. The Research Ethics Committee of the Hospital District of Northern Savo approved the study protocol in 2006 (statement 69/2006). The parents or caregivers of the children gave their written informed consent, and the children provided their assent to participation. The PANIC study has been performed in accordance with the principles of the Declaration of Helsinki as revised in 2008.

### Assessment of Cardiac Work and Function

Stroke volume (milliliters), cardiac output (liters per minute), and cardiac work (kilograms×meters) were measured after a 15‐minute supine rest with the CircMon B202 impedance cardiography device (JR Medical, Saku Vald, Estonia) coupled with the Finapress device.^26^, ^27^ Cardiac work reflects the work performed by the left ventricle and is closely correlated with cardiac oxygen demand.^17^ These measures were also normalized for body surface area and expressed as stroke index (milliliters per meters squared), cardiac index (liters per minute per meters squared), and cardiac work index (kilograms×meters per meters squared). A higher stroke index, cardiac index, and cardiac work index reflect higher stroke volume, cardiac output, and left ventricle workload, respectively. The method and electrode configuration (Figure S1) have been described in detail elsewhere.^26^ Cardiac work index was calculated using the formula:

$$\begin{array}{c} 0.0143\times (\text{mean arterial pressure}-\text{pulmonary artery}\\ \;\text{occlusion pressure})\times \text{cardiac index}. \end{array}$$

Pulmonary artery occlusion pressure was assumed to be 6 mm Hg, and 0.0143 is the conversion factor of pressure from millimeters of mercury to centimeters of water, volume to density of blood (in kilograms per liter), and centimeters to meters.^27^ Beat‐to‐beat blood pressure used to assess mean arterial pressure was measured using the Finapress device and was analyzed using the Cafts program. Cardiac output measured using the CircMon whole‐body impedance cardiography agree reasonably well with 3‐dimensional ultrasound measurement and the thermodilution method.^26^, ^28^, ^29^ Impedance cardiography has also shown reasonable validity and reproducibility in youth.^30^, ^31^, ^32^

### Assessment of Sedentary Time and Physical Activity

A uniaxial accelerometer with a built‐in heart rate sensor (Actiheart; CamNtech, Papworth, United Kingdom) was attached to the chest via ECG electrodes and used to assess sedentary time and PA. The device was set to record body movement and heart rate in 60‐second epochs. The participants were instructed to carry on with their usual behavior and to wear the monitor during all daily activities, including sleep, shower, sauna, and swimming, as described previously.^33^, ^34^ The participants were therefore requested to wear the device continuously for a minimum of 4 consecutive days, 2 on weekdays and 2 on the weekend, because the activity patterns of school children are known to vary markedly between weekdays and weekend days.^35^ At baseline, the median monitor wear time was 104 hours (minimum–maximum 52–212 hours), at 2‐year follow‐up 101 hours (48–171 hours), and at 8‐year follow‐up 170 hours (65–425 hours). We accepted sedentary time and PA data for statistical analyses if there were at least 48 hours of activity recording in weekday and weekend day hours that included at least 12 hours from morning (3 am–9 am), noon (9 am–3 pm), afternoon (3 pm–9 pm), and night (9 pm–3 am) to avoid potential bias from overrepresenting specific times and activities of the days.^36^ This resulted in at least 12 hours of wear data from morning (3 am–9 am), noon (9 am–3 pm), afternoon/evening (3 pm–9 pm), and night (9 pm–3 am).

Upon retrieving and downloading the data from the device, heart rate data were first corrected for noise.^37^ Subsequently, they were individually calibrated with sleeping heart rate and parameters obtained from maximal exercise tests^38^, ^39^ performed by the Ergoselect 200 K electromagnetic bicycle ergometer (Ergoline, Bitz, Germany) and the Cardiosoft V6.5 Diagnostic System ECG device (GE Healthcare Medical Systems, Freiburg, Germany). The heart rate data were finally combined with trunk acceleration data in a branched equation model to estimate activity intensity time series.^40^ Monitor nonwear was acknowledged by prolonged 0 acceleration lasting >90 minutes accompanied by nonphysiological heart rate, and activity estimates were adjusted during summarization to minimize diurnal bias arising from nonwear. PA energy expenditure (PAEE) was calculated by integrating the intensity time series, where time distribution of activity intensity was generated by using standard metabolic equivalent tasks (METs) in 0.5 increments. Sleep duration was analyzed from the Actiheart recordings by a trained exercise specialist and confirmed by a physician, where necessary.^34^ The time of falling asleep was defined as accelerometer counts decreasing to 0 and heart rate to a plateau level. The time of waking up was defined as accelerometer counts increasing and remaining >0 and heart rate increasing and remaining above the plateau level. We defined sedentary time as time spent in activity ≤1.5 METs excluding sleep and light PA, moderate PA, and vigorous PA as time spent in activity >1.5 and ≤4.0 METs, >4.0 and ≤7.0 METs, and >7.0 METs, respectively, by defining 1 MET as 71.2 J/min per kilogram. MVPA included moderate PA and vigorous PA. The cutoffs have been commonly applied in investigations of PA among children and youth. Accelerometers with built‐in heart rate monitoring capabilities have been found to be more accurate in estimating PAEE than either method alone in children,^41^, ^42^ explaining 86% of variance in PA energy expenditure variance.^42^

We used the area under the curve (AUC) approach for sedentary time and PA measured at baseline, 2‐year follow‐up, and 8‐year follow‐up to use all of the data collected over the 8‐year period and to describe the exposure to sedentary time and PA from childhood to adolescence.^43^ The AUCs were determined using an additive mixed model.^44^ The modeling allowed the inclusion of a nonlinear effect that was modeled by cubic spline in addition to random intercept for individuals.^45^ For this study, the AUC variables for sedentary time and PA were defined separately for childhood (from baseline to 2‐year follow‐up) and from childhood to adolescence (from baseline to 8‐year follow‐up).

Because the AUC approach used to quantify cumulative sedentary time and PA uses estimated data, we also performed the AUC analyses among 81 participants (28 girls, 53 boys) who had valid and complete data on sedentary time and PA at all 3 time points. We performed additional supplemental analyses using mean sedentary time and PA from baseline to 2‐year follow‐up and from baseline to 8‐year follow‐up among 81 participants (28 girls, 53 boys) who had valid and complete data for sedentary time and PA in all time points. This was performed to account for the reduction in the reliability of the data due to the long interval from the assessment of sedentary time and PA from childhood to adolescence.^46^

### Assessment of Modifying Factors

Whole body mass was measured twice, with the children having fasted for 12 hours, emptied the bladder, and standing in light underwear by a calibrated InBody 720 bioelectrical impedance device (Biospace, Seoul, South Korea) to an accuracy of 0.1 kg. The mean of these 2 values was used in the analyses. Stature was measured 3 times with the children standing in the Frankfurt plane without shoes using a wall‐mounted stadiometer to an accuracy of 0.1 cm. The mean of the nearest 2 values was used in the analyses. Body mass index was calculated by dividing body mass (kilograms) by body height (meters squared). Body mass index–SD score was calculated based on Finnish reference data.^47^ The prevalence of overweight and obesity was defined using the cutoff values provided by Cole et al.^48^ Total fat mass and body fat percentage (BF%) were measured by the Lunar dual‐energy x‐ray absorptiometry device (GE Medical Systems, Madison, WI) using standardized protocols.^49^

Systolic blood pressure was measured from the right arm using the Heine Gamma G7 aneroid sphygmomanometer (Heine Optotechnik) to an accuracy of 2 mm Hg. The measurement protocol included a 5‐minute seated resting period followed by 3 measurements with 2‐minute intervals in between. The average of all 3 values was used in the analyses. Pulse wave velocity (PWV) was measured with the CircMon B202 impedance cardiography device (JR Medical, Saku Vald, Estonia). The participants were asked to rest for 15 minutes in a supine position before the measurement. The CircMon software estimates the foot of the impedance cardiography signal that coincides with pulse transmission in the aortic arch. The distal impedance plethysmogram was recorded from the popliteal artery at the knee joint level. Using the measured pulse transit time (Δ*t*) and assessed distance (*L*) between these 2 sites, the CircMon software calculates PWV using the equation: PWV (m/s)=*L*/Δ*t*.^50^

A research nurse took blood samples in the morning, after children had fasted overnight for at least 12 hours. Blood was immediately centrifuged and stored at a temperature of −75 °C until biochemical analyses. Plasma glucose was measured by a hexokinase method, and serum insulin was measured by an electrochemiluminescence immunoassay. Intra‐assay and interassay coefficient of variation for the insulin analyses were 1.3% to 3.5% and 1.6% to 4.4%, respectively. Insulin resistance was assessed using Homeostatic Model Assessment for Insulin Resistance and the formula^51^:

$$\frac{\text{fasting serum insulin}\times \text{fasting plasma glucose}}{22.5}.$$

The Nightingale high‐throughput nuclear magnetic resonance spectroscopy platform was used to assess HDL cholesterol, average HDL diameter, and the concentrations of extra‐large, large, medium, and small HDL particles, and the concentration of apolipoprotein A1.^52^

A research physician assessed pubertal status using a 5‐stage scale described by Marshall and Tanner.^53^, ^54^ We used testicular volume assessed by an orchidometer to assess pubertal status in boys and breast development to assess pubertal status in girls.

### Statistical Analysis

Statistical analyses were performed using the SPSS statistical software, version 28.0 (IBM, Armonk, NY). The characteristics of participants were presented as means (SDs) or medians (interquartile ranges), or percentages for normally distributed continuous variables, continuous variables with skewed distributions, or categorical variables, respectively. The characteristics between those included in the analyses and those excluded were compared using the Student *t* test for normally distributed continuous variables, the Mann‐Whitney *U* test for continuous variables with skewed distributions, or the χ^2^ test for categorical variables. Before the analyses, we performed square root or natural logarithm transformation for skewed variables. The associations of cumulative sedentary time and PA with the measures of cardiac work and function were investigated using linear regression analyses adjusted for age and sex. The data were corrected for multiple comparisons using the Benjamini‐Hochberg false discovery rate using the false discovery rate value of 0.1. These data were further adjusted for possible modifiers of the associations including pubertal status, BF%, Homeostatic Model Assessment for Insulin Resistance, systolic blood pressure, arterial stiffness, or HDL characteristics, which were entered into the models separately. We replaced the missing data on these measures by multiple imputation using 10 imputed data sets. To study the modifying effect of sex on the associations of cumulative sedentary time and PA with measures of cardiac work and function, we included a sex×sedentary time or sex×PA interaction term in the models. For the current 3 predictor analyses, we estimated that 121 observations were needed to observe a small effect size (*f* ^2^=0.05) at the power of 0.80 when statistical significance level was set at *P*<0.05.^55^ We considered standardized regression coefficients between 0.10 and 0.29, between 0.30 and 0.49, and ≥0.50 to describe small, medium, and large effect sizes, respectively.^55^

## RESULTS

### Participants

**Figure 1:**
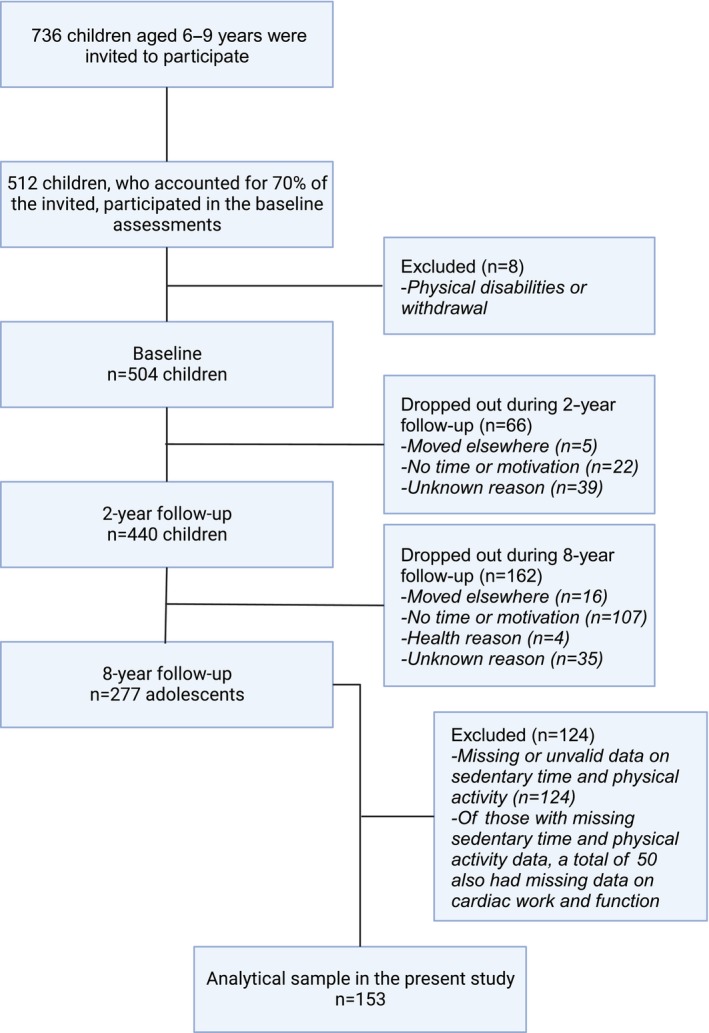
Flowchart of the study.

Altogether, 736 children aged 6 to 8 years from primary schools of Kuopio were invited to participate in the baseline examination in 2007 to 2009. A total of 512 children, who represented 70% of those invited, participated in the baseline examinations. Six children were excluded from the study at baseline because of physical disabilities that could hamper participation in the intervention or had no time or motivation to attend the study. Based on data from the Finnish national school health examinations, the participants did not differ in sex distribution, age, or body mass index–SD score from all other children who started the first grade in 2007 to 2009. We conducted the main analyses for 153 participants (69 girls, 84 boys) who had valid data on cardiac work and function at 8‐year follow‐up and valid sedentary time and PA data at least at 1 time point (Figure 1). Participants included in the analyses did not differ in age, sex distribution, pubertal status, body mass index–SD score, or BF% at 8‐year follow‐up from those excluded from the analyses (all *P*>0.160).

### Characteristics of Participants

The participants' characteristics at 8‐year follow‐up are presented in the Table 1. In general, girls had more advanced pubertal development, higher BF%, and accumulated less MVPA and vigorous PA than boys.

**Table 1:** Basic Characteristics

Characteristic	All	Girls	Boys
Age, y	15.8 (15.5–16.1)	15.8 (15.4–16.1)	15.8 (15.5–16.2)
Height, cm	171.9 (8.1)	166.6 (5.4)	176.3 (7.2)
Weight, kg	59.5 (54.6–67.0)	57.2 (52.7–63.1)	63.3 (56.2–73.2)
BMI	20.6 (18.8–22.0)	20.9 (19.4–21.9)	20.1 (18.5–22.1)
BMI‐SDS	0.03 (0.9)	0.16 (0.9)	−0.08 (1.0)
Prevalence of overweight/obesity (%)	15.7	14.5	16.7
Pubertal status (%)			
3	7.5	1.5	12.7
4	56.8	52.2	60.8
5	35.6	46.3	26.6
Body fat percentage	24.2 (13.2–30.6)	29.5 (24.6–34.3)	14.6 (10.7–22.8)
HOMA‐IR (n=143)	2.2 (1.7–3.1)	2.3 (1.8–3.1)	2.1 (1.6–3.3)
Systolic blood pressure, mm Hg	113 (11)	110 (8)	116 (12)
Pulse wave velocity (m/s) (n=145)	5.7 (5.5–6.1)	5.7 (5.5–6.1)	5.7 (5.4–6.0)
HDL cholesterol (mmol/L) (n=148)	1.4 (0.2)	1.5 (0.2)	1.2 (0.2)
Concentration of HDL particles (mmol/L) (n=148)	0.015 (0.001)	0.016 (0.002)	0.014 (0.001)
Average HDL diameter (nm) (n=148)	9.7 (0.2)		
Concentration of extra‐large HDL particles (mmol/L) (n=148)	0.0002 (0.0002–0.0003)	0.0002 (0.0002–0.0003)	0.0002 (0.0001–0.0002)
Concentration of large HDL particles (mmol/L) (n=148)	0.001 (0.001–0.002)	0.002 (0.001–0.002)	0.001 (0.001–0.002)
Concentration of medium HDL particles (mmol/L) (n=148)	0.004 (0.001)	0.004 (0.001)	0.003 (0.001)
Concentration of small HDL particles (mmol/L) (n=148)	0.009 (0.001)	0.010 (0.001)	0.009 (0.001)
Apolipoprotein A1 (g/L) (n=148)	1.4 (0.18)	1.5 (0.18)	1.3 (0.13)
Sedentary time, min/d	604 (139)	609 (142)	601 (139)
Light physical activity, min/d	321 (117)	321 (124)	322 (113)
Moderate to vigorous physical activity, min/d	39 (20–68)	25 (19–45)	51 (26–71)
Vigorous physical activity, min/d	5 (1–19)	2 (0–6)	9 (1–26)
Physical activity energy expenditure, kJ/kg per d	55.9 (22.5)	51.9 (19.6)	59.1 (24.3)
Cardiac work, kg×m	5.1 (1.3)	4.9 (1.1)	5.2 (1.4)
Cardiac work index, kg×m/m^2^	2.9 (0.7)	3.0 (0.7)	2.9 (0.7)
Stroke volume index, mL/m^2^	42.1 (6.3)	42.2 (6.1)	42.1 (6.6)
Cardiac index, L/min per m^2^	2.9 (0.5)	3.0 (0.5)	2.9 (0.5)

The data are means and SDs or medians and interquartile ranges. *P* values for the differences between girls and boys are from the Student *t* test, Mann‐Whitney *U* test, or χ^2^ test. BMI indicates body mass index; BMI‐SDS, body mass index–SD score; HDL, high‐density lipoprotein; and HOMA‐IR, Homeostatic Model Assessment for Insulin Resistance.

### Associations of Cumulative Sedentary Time With Cardiac Work and Function

**Figure 2:**
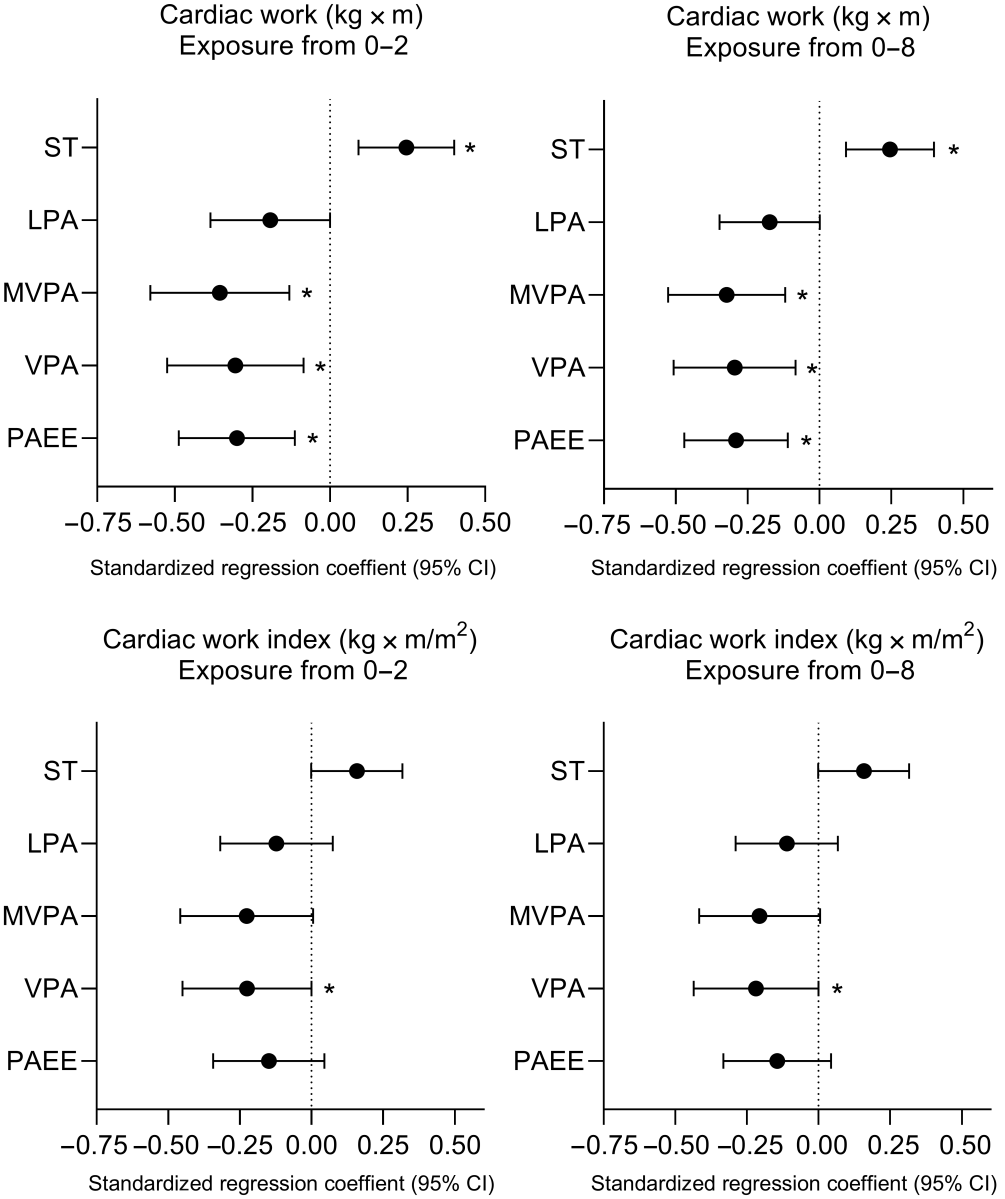
Associations of indices of cumulative sedentary time and physical activity from baseline to 2‐year follow‐up and from baseline to 8‐year follow‐up with cardiac work and cardiac work index at 8‐year follow‐up. Data are standardized regression coefficients with their 95% CIs adjusted for age and sex. Exposure from 0 to 2 denotes cumulative sedentary time or physical activity from baseline to 2‐year follow‐up, and exposure from 0 to 8 denotes cumulative sedentary time or physical activity from baseline to 8‐year follow‐up. *P<0.05. LPA indicates light physical activity; MVPA, moderate‐to‐vigorous physical activity; PAEE, physical activity energy expenditure; ST, sedentary time; and VPA, vigorous physical activity.

A cumulative sedentary time from baseline to 2‐year follow‐up and from baseline to 8‐year follow‐up was directly associated with cardiac work at 8‐year follow‐up after adjustment for age and sex (Figure 2, Table S1). These associations remained also after false discovery rate correction of 0.1.

#### Effect of Modifying Factors on the Associations of Cumulative Sedentary Time With Cardiac Work and Function

The associations of cumulative sedentary time from baseline to 2‐year follow‐up (β=0.154 [95% CI, −0.005 to 0.312]) and from baseline to 8‐year follow‐up (β=0.153 [95% CI, −0.005 to 0.310]) with cardiac work attenuated after further adjustment for BF%.

#### Modifying Effect of Sex on the Associations of Cumulative Sedentary Time With Cardiac Work and Function

Higher cumulative sedentary time from baseline to 2‐year follow‐up and from baseline to 8‐year follow‐up were associated with lower stroke volume index in girls (β=−0.257 [95% CI, −0.496 to −0.018]) but not in boys (β=0.178 [95% CI, −0.038 to 0.394], *P*=0.009 for interaction). Further adjustment for PWV attenuated the inverse association between sedentary time and stroke volume index in girls (β=−0.213 [95% CI, −0.458 to 0.031]). In these analyses, the standardized regression coefficients were identical for both time points.

### Associations of Cumulative Physical Activity With Cardiac Work and Function

Cumulative MVPA and PAEE from baseline to 2‐year follow‐up and from baseline to 8‐year follow‐up were inversely associated with cardiac work and cardiac index after adjustment for age and sex (Figure 2, Table S1). Cumulative vigorous PA from baseline to 2‐year follow‐up and from baseline to 8‐year follow‐up was inversely associated with cardiac work and cardiac work index. These associations also remained after false discovery rate 0.1 correction.

#### Effect of Modifying Factors on the Associations of Cumulative Physical Activity With Cardiac Work and Function

Further adjustment for BF% at 8‐year follow‐up attenuated the associations of cumulative MVPA (β=−0.205 [95% CI, −0.439 to 0.029] and β=−0.187 [95% CI, −0.400 to 0.027] for cumulative exposures from baseline to 2‐year‐follow‐up and from baseline to 8‐year follow‐up, respectively), cumulative vigorous PA (β=−0.171 [95% CI, −0.385 to 0.054] and β=−0.165 [95% CI, −0.382 to 0.468]), and cumulative PAEE (β=−0.163 [95% CI, −0.363 to 0.037] and β=−0.158 [95% CI, −0.351 to 0.035]) with cardiac work.

The association between cumulative vigorous PA and cardiac work index attenuated after further adjustment for BF% at 8‐year follow‐up (β=−0.203 [95% CI, −0.443 to 0.036] and β=−0.197 [95% CI, −0.429 to 0.035]), Homeostatic Model Assessment for Insulin Resistance at 8‐year follow‐up (β=−0.225 [95% CI, −0.451 to 0.002] and β=−0.218, [95% CI, −0.437 to 0.001]), or PWV at 8‐year follow‐up (β=−0.191 [95% CI, −0.141 to 0.033] and β=−0.185 [95% CI, −0.413 to 0.031]).

The associations of cumulative MVPA (β=−0.217 [95% CI, −0.448 to 0.014] and β=−0.198 [95% CI, −0.408 to 0.013]) and cumulative PAEE (β=−0.181 [95% CI, −0.374 to 0.011] and β=−0.175 [95% CI, −0.362 to 0.011]) with cardiac index attenuated after further adjustment for PWV at 8‐year follow‐up.

#### Modifying Effect of Sex on the Associations of Cumulative Physical Activity With Cardiac Work and Function

Cumulative light PA (β=0.257 [95% CI, 0.020–0.494] versus β=−0.120 [95% CI, −0.338 to 0.097], *P*=0.026 for interaction) and cumulative vigorous PA (β=0.237 [95% CI, 0.000–0.474] versus β=−0.048 [95% CI, −0.367 to 0.171], *P*=0.059 for interaction) from baseline to 2‐year follow‐up and from baseline to 8‐year follow‐up were directly associated with stroke volume index in girls but not in boys. In these analyses, the standardized regression coefficients were identical for both time points.

### Supplemental Analyses

Cumulative sedentary time from baseline to 2‐year follow‐up and from baseline to 8‐year follow‐up was directly associated with cardiac work, cardiac work index, and cardiac index at 8‐year follow‐up (Table S2). Cumulative MVPA from baseline to 2‐year follow‐up and from baseline to 8‐year follow‐up was inversely associated with cardiac work. A higher PAEE from baseline to 2‐year follow‐up and from baseline to 8‐year follow‐up was associated with lower cardiac work, cardiac work index, and cardiac index.

A higher mean sedentary time from baseline to 8‐year follow‐up was associated with higher cardiac work, cardiac work index, and cardiac index (Table S3). A higher mean MVPA from baseline to 8‐year follow‐up was inversely associated with cardiac work. Average PAEE from baseline to 2‐year follow‐up and from baseline to 8‐year follow‐up was associated with lower cardiac work. A higher average PAEE from baseline to 8‐year follow‐up was associated with lower cardiac work index and cardiac index.

Further adjustment for BF%, Homeostatic Model Assessment for Insulin Resistance, and PWV at 8‐year follow‐up attenuated the association between MVPA and cardiac work. Further adjustment for systolic blood pressure and PWV at 8‐year follow‐up attenuated the associations of sedentary time or PAEE with cardiac work index, respectively.

## DISCUSSION

We observed that higher cumulative sedentary time and lower cumulative MVPA, vigorous PA, and PAEE in childhood and from childhood to adolescence were associated with higher cardiac work in adolescence. However, only cumulative vigorous PA was inversely associated with the cardiac work index. Nevertheless, these associations of sedentary time and PA with cardiac work attenuated after controlling for BF%. Interestingly, when we applied tighter inclusion criteria for sedentary time and PA data in the supplemental analyses, lower levels of sedentary time and higher PAEE were associated with lower cardiac work index independent of BF% but not systolic blood pressure or PWV. Therefore, our findings suggest that higher cumulative sedentary time and lower cumulative PA in childhood and from childhood to adolescence are associated with higher cardiac work in adolescence. Furthermore, the effect sizes for all associations were considered small to medium.

Our findings suggest that higher levels of sedentary time are associated with higher cardiac work and cardiac oxygen demand. Higher levels of sedentary time have been associated with higher left ventricular mass in adolescents^15^ and adults,^10^ indicating possible pathophysiological alterations underlying left ventricular hypertrophy and higher cardiac oxygen consumption.^16^ As such, the enlargement in cardiac muscle and left ventricular size may explain the present findings of a direct association between sedentary time and cardiac work. On the other hand, MVPA has also been positively associated with a left ventricular mass in adolescents.^15^ However, this could be attributed to a positive cardiac adaptation known as athlete's heart. Long‐term endurance training increases left ventricular mass in youth^56^ and adults^57^ and reduces left ventricular wall stress, thereby decreasing cardiac oxygen demand.^58^ This is supported by the inverse relationship we found between cumulative vigorous PA and cardiac work index.

In line with previous studies in adults,^13^ we found that higher levels of PA were associated with lower cardiac work. Moreover, our current observations suggest that at least moderate‐intensity PA, but not light‐intensity PA, was inversely associated with cardiac work. It is likely that similar to adults, an intensity threshold for PA in youth needs to be met before beneficial cardiac adaptations^59^ occur. These findings align with observations that higher‐intensity PA is more effective in improving cardiovascular function, such as maximal oxygen uptake,^60^ and endothelial function in youth.^61^ Nevertheless, we observed an inverse association between PAEE and cardiac work, suggesting that the total volume of PA with sufficient intensity is an important determinant of cardiac work as well.

Our findings, in line with the results of some previous studies,^58^, ^62^ suggest that sedentary time and PA have weak, if any, associations with resting stroke volume and cardiac output at best. Nevertheless, we observed that sedentary time was directly and PAEE was inversely associated with cardiac index. These results could be related to alterations in cardiac structures and cardiovascular regulation.^10^, ^15^, ^58^ However, differences in resting heart rate are the most plausible explanation for these observations, because youth with higher levels of sedentary time and lower PAEE have higher resting heart rates directly contributing to higher cardiac output.

The associations between sedentary time, PA, and cardiac work were attenuated after controlling for BF% and may partially explain the observed associations of sedentary time and PA with cardiac work. Obesity increases the cardiac work levels^63^ and has a tightly interwoven relationship with increased sedentary time in youth.^64^, ^65^ Moreover, decrease in PA levels has also been documented to mediate body mass gain in adolescents.^66^ As such, the independent role of sedentary time in the alterations of cardiac work and function remains to be completely established.^10^ Notwithstanding, in adults with obesity, weight loss has been associated with reduced left ventricular mass.^67^ Therefore, preventing increased adiposity, insulin resistance, and arterial stiffness by reducing sedentary time and increasing PA may reduce cardiac workload in youth.

Multicomponent interventions targeting several unhealthy behaviors and cardiometabolic risk factors may have more usefulness in preventing cardiovascular diseases from the time of youth.^68^ Accordingly, it has been suggested that cardiovascular health is largely influenced by sedentary behaviors, PA, diet quality, environment, sleep, and mental well‐being.^69^ Therefore, a combination of reducing sedentary time, increasing PA, and improving diet and sleep quality from childhood should be the first line of cardiovascular disease prevention.^70^, ^71^, ^72^ In addition, reducing environmental pollution^73^ and mental health disorders^74^ are also critical components for cardiovascular health improvement and the prevention of cardiovascular disease risk in youth.

The strengths of the present study include an 8‐year follow‐up among a relatively large sample of adolescents using valid and reproducible methods to assess sedentary time, PA, and cardiac work and function. We also controlled for several possible confounding factors, allowing us to investigate their role in the associations of sedentary time and PA with cardiac work and function. However, due to missing data on sedentary time and PA at the 8‐year follow‐up stage, data from 45% of the participants were omitted from the analyses. Furthermore, the sample size was also relatively small for sex‐specific associations, which decreased the statistical power to detect significant associations. There are also several other limitations that need to be noted. Firstly, the nature of the study means that causality cannot be ascertained. Secondly, we assessed cardiac work and function only in adolescence and not throughout childhood to adolescence. Thirdly, we were not able to assess the role of posture or sit‐to‐stand transitions or prolonged sitting in cardiac work and function, although they have been associated with cardiovascular health in adults.^75^ Moreover, sedentary time assessed by the Actiheart monitor may also underestimate the time spent in sedentary behaviors,^76^, ^77^ because it is placed on the chest as opposed to being on the thigh, which would provide a more accurate assessment. In addition, indices of cumulative sedentary time and PA from baseline to 8‐year follow‐up do not capture possible fluctuations in sedentary time and PA between 2‐year and 8‐year follow‐up and therefore may over‐ or underestimate overall exposure to sedentary time and PA in some participants.

Finally, we are aware that cardiac magnetic resonance imaging or echocardiography, as the gold standard methods to measure stroke volume and cardiac output, could have increased the validity of our findings. However, stroke volume and cardiac output measured using impedance cardiography show a reasonable level of agreement with other methods.^26^, ^28^, ^29^ Moreover, the easily available assessment of cardiac work using impedance cardiography could have clinical value, because increased cardiac workload may increase the risk of heart failure.^16^ Regardless, there are no clinical thresholds for increased cardiac workload for pediatric populations, and therefore the interpretations should be made cautiously. Our sample included apparently healthy White adolescents, and therefore our findings may not be directly generalizable to other populations. Nevertheless, the levels of sedentary time and PA in our study sample were comparable to those of larger study population of Finnish adolescents.^1^

In conclusion, higher cumulative sedentary time, lower cumulative MVPA, and cumulative vigorous PA from middle childhood to adolescence were associated with higher cardiac work in adolescence. However, adiposity and other cardiometabolic risk factors partially explain these associations. Our results highlight the importance of increasing PA and obesity prevention from childhood to decrease the risk of cardiac‐related abnormalities later in life. Long‐term observational and intervention studies are required to establish the role of PA increase and sedentary time reduction in cardiac functions in children and adolescents, and to investigate possible sex‐differences in the associations and effects.

## Acknowlegments

Author contributions: E.A.H., conceptualization, formal analysis, writing original draft, funding acquisition. M.H.L., conceptualization, writing review and editing. E.L., writing review and editing. K.S., methodology, investigation, writing review and editing. J.A.L., methodology, investigation, writing review and editing. M.K., methodology, writing review and editing. S.B., methodology, writing review and editing. TAL, supervision, funding acquisition, project administration, methodology, investigation, writing review and editing. All authors approved the final version of the article.

## Sources of Funding

The PANIC study has been supported financially by grants from the Research Council of Finland, Ministry of Education and Culture of Finland, Ministry of Social Affairs and Health of Finland, Research Committee of the Kuopio University Hospital Catchment Area (State Research Funding), Finnish Innovation Fund Sitra, Social Insurance Institution of Finland, Finnish Cultural Foundation, Foundation for Pediatric Research, Diabetes Research Foundation in Finland, Finnish Foundation for Cardiovascular Research, Juho Vainio Foundation, Paavo Nurmi Foundation, Yrjö Jahnsson Foundation, and the city of Kuopio.

## Disclosures

None.

## Supporting information

Tables S1–S3
Figure S1
