# Supplementary material for: Accumulating Sedentary Time and Physical Activity From Childhood to Adolescence and Cardiac Function in Adolescence
Source: J Am Heart Assoc. 2024 Mar 16;13(6):e031837. doi: 10.1161/JAHA.123.031837 (PMC11010014; doi:10.1161/JAHA.123.031837)

# SUPPLEMENTAL MATERIAL

**Table S1. Associations of cumulative sedentary time and physical activity with cardiac work and function in 153 adolescents.**

| | Left cardiac work (kg x m) | Left cardiac work index (kg x m/m$^2$) | Stroke volume index (mL/m$^2$) | Cardiac index (mL/min/m$^2$) |
|---|---|---|---|---|
| Sedentary time | | | | |
| 0–2 | **0.246 (0.092 to 0.400)** | 0.158 (-0.001 to 0.317) | -0.003 (-0.164 to 0.158) | 0.143 (-0.016 to 0.302) |
| 0–8 | **0.245 (0.092 to 0.398)** | 0.158 (-0.001 to 0.316) | -0.003 (-0.164 to 0.158) | 0.142 (-0.016 to 0.301) |
| Light physical activity | | | | |
| 0–2 | -0.192 (-0.385 to 0.001) | -0.123 (-0.319 to 0.074) | 0.066 (-0.132 to 0.263) | -0.094 (-0.291 to 0.103) |
| 0–8 | -0.174 (-0.348 to 0.001) | -0.111 (-0.289 to 0.067) | 0.059 (-0.120 to 0.239) | -0.085 (-0.263 to 0.093) |
| Moderate-to-vigorous physical activity | | | | |
| 0–2 | **-0.355 (-0.579 to -0.131)** | -0.226 (-0.458 to 0.005) | -0.070 (-0.304 to 0.165) | **-0.251 (-0.481 to -0.021)** |
| 0–8 | **-0.323 (-0.527 to -0.119)** | -0.206 (-0.417 to 0.005) | -0.063 (-0.277 to 0.150) | **-0.229 (-0.439 to -0.019)** |
| Vigorous physical activity | | | | |
| 0–2 | **-0.305 (-0.524 to -0.085)** | **-0.225 (-0.450 to 0.000)** | 0.043 (-0.185 to 0.271) | -0.164 (-0.389 to 0.062) |
| 0–8 | **-0.295 (-0.508 to -0.083)** | **-0.218 (-0.436 to 0.000)** | 0.042 (-0.179 to 0.263) | -0.159 (-0.377 to 0.060) |
| Physical activity energy expenditure | | | | |
| 0–2 | **-0.300 (-0.487 to -0.113)** | -0.149 (-0.343 to 0.045) | -0.067 (-0.263 to 0.129) | **-0.209 (-0.401 to -0.017)** |
| 0–8 | **-0.290 (-0.471 to -0.110)** | -0.144 (-0.332 to 0.044) | -0.065 (-0.254 to 0.124) | **-0.202 (-0.388 to -0.016)** |

The data are standardised regression coefficients and their 95% confidence intervals adjusted for age and sex. Statistically significant associations are bolded. 0–2 and 0–8 describe time points used to calculated cumulative exposures.

**Table S2. Associations of cumulative sedentary time and physical activity with cardiac work and function in 81 adolescents.**

| | Left cardiac work (kg x m) | Left cardiac work index (kg x m/m$^2$) | Stroke volume index (mL/m$^2$) | Cardiac index (mL/min/m$^2$) |
|---|---|---|---|---|
| Sedentary time | | | | |
| 0–2 | **0.288 (0.080 to 0.496)** | **0.231 (0.013 to 0.450)** | 0.081 (-0.144 to 0.306) | **0.248 (0.028 to 0.468)** |
| 0–8 | **0.286 (0.080 to 0.492)** | **0.230 (0.013 to 0.447)** | 0.081 (-0.142 to 0.304) | **0.246 (0.028 to 0.465)** |
| Light physical activity | | | | |
| 0–2 | -0.192 (-0.430 to 0.047) | -0.168 (-0.416 to 0.079) | -0.063 (-0.314 to 0.187) | -0.208 (-0.457 to 0.040) |
| 0–8 | -0.178 (-0.400 to 0.044) | -0.156 (-0.386 to 0.074) | -0.059 (-0.292 to 0.174) | -0.193 (-0.424 to 0.037) |
| Moderate-to-vigorous physical activity | | | | |
| 0–2 | **-0.333 (-0.029 to 0.629)** | -0.273 (-0.583 to 0.037) | -0.063 (-0.380 to 0.254) | -0.296 (-0.608 to 0.016) |
| 0–8 | **-0.304 (-0.576 to -0.033)** | -0.250 (-0.534 to 0.034) | -0.058 (-0.348 to 0.232) | -0.271 (-0.557 to 0.014) |
| Vigorous physical activity | | | | |
| 0–2 | -0.248 (-0.525 to 0.030) | -0.155 (-0.446 to 0.135) | -0.036 (-0.330 to 0.257) | -0.152 (-0.446 to 0.142) |
| 0–8 | -0.241 (-0.512 to -0.029) | -0.151 (-0.434 to 0.132) | -0.035 (-0.321 to 0.250) | -0.148 (-0.434 to 0.138) |
| Physical activity energy expenditure | | | | |
| 0–2 | **-0.374 (-0.630 to -0.118)** | **-0.298 (-0.569 to -0.028)** | -0.085 (-0.364 to 0.194) | **-0.327 (-0.599 to -0.056)** |
| 0–8 | **-0.362 (-0.610 to -0.114)** | **-0.289 (-0.550 to -0.027)** | -0.082 (-0.352 to 0.187) | **-0.317 (-0.579 to -0.054)** |

The data are standardised regression coefficients and their 95% confidence intervals adjusted for age and sex. Statistically significant associations are bolded. 0–2 and 0–8 describe time points used to calculated cumulative exposures.

**Table S3. Associations of mean sedentary time and physical activity with cardiac work and function in 81 adolescents.**

| | Left cardiac work (kg x m) | Left cardiac work index (kg x m/m$^2$) | Stroke volume index (mL/m$^2$) | Cardiac index (mL/min/m$^2$) |
|---|---|---|---|---|
| Sedentary time | | | | |
| 0–2 | 0.179 (-0.031 to 0.390) | 0.116 (-0.104 to 0.336) | 0.122 (-0.099 to 0.342) | 0.100 (-0.123 to 0.322) |
| 0–8 | **0.284 (0.079 to 0.489)** | **0.228 (0.012 to 0.444)** | 0.080 (-0.141 to 0.302) | **0.245 (0.028 to 0.462)** |
| Light physical activity | | | | |
| 0–2 | -0.056 (-0.276 to 0.163) | -0.015 (-0.242 to 0.212) | 0.113 (-0.339 to 0.114) | -0.051 (-0.281 to 0.178) |
| 0–8 | -0.171 (-0.383 to 0.042) | -0.150 (-0.370 to 0.071) | 0.056 (-0.279 to 0.167) | -0.185 (-0.406 to 0.036) |
| Moderate-to-vigorous physical activity | | | | |
| 0–2 | -0.188 (-0.416 to 0.039) | -0.151 (-0.388 to 0.085) | -0.045 (-0.284 to 0.195) | -0.130 (-0.370 to 0.110) |
| 0–8 | **-0.254 (-0.480 to -0.027)** | -0.208 (-0.444 to 0.028) | -0.048 (-0.289 to 0.194) | -0.226 (-0.464 to 0.012) |
| Vigorous physical activity | | | | |
| 0–2 | -0.188 (-0.416 to 0.039) | -0.151 (-0.388 to 0.085) | 0.045 (-0.284 to 0.195) | -0.130 (-0.370 to 0.110) |
| 0–8 | -0.204 (-0.402 to 0.025) | -0.128 (-0.367 to 0.111) | 0.048 (-0.289 to 0.194) | -0.226 (-0.464 to 0.012) |
| Physical activity energy expenditure | | | | |
| 0–2 | **-0.260 (-0.481 to -0.038)** | -0.193 (-0.425 to 0.040) | -0.099 (-0.335 to 0.138) | -0.179 (-0.414 to 0.057) |
| 0–8 | **-0.321 (-0.540 to -0.101)** | **-0.256 (-0.487 to -0.024)** | -0.073 (-0.312 to 0.166) | **-0.280 (-0.513 to -0.048)** |

The data are standardised regression coefficients and their 95% confidence intervals adjusted for age and sex. Statistically significant associations are bolded. 0–2 and 0–8 describe time points used to calculated mean exposures.

**Figure S1. Electrode placement for the impedance cardiography measurement.**

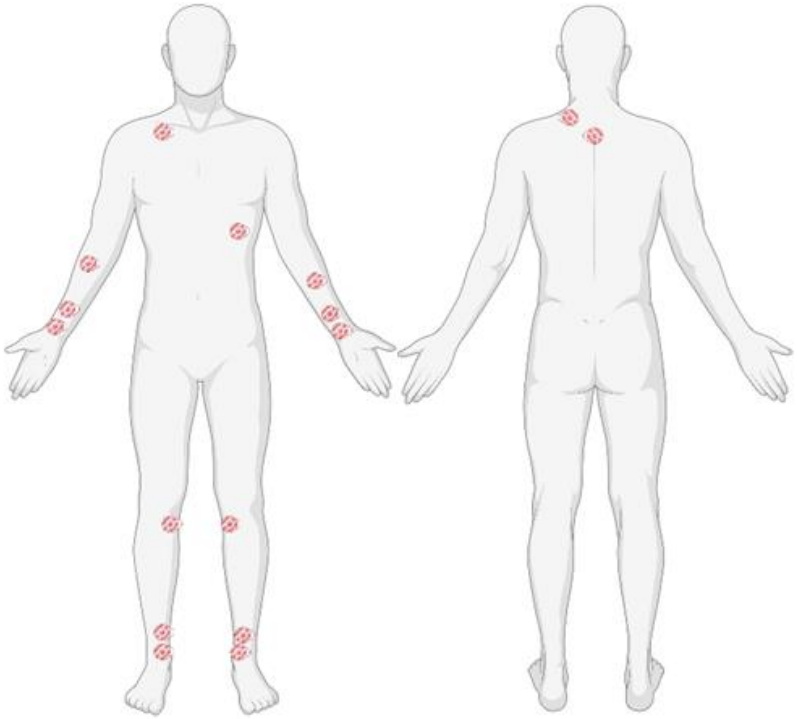

Supplement: Supplementary file 1 — Tables S1–S3 Figure S1 [file JAH3-13-e031837-s001.pdf]
